# Catalytic Performance of Nitrogen-Doped Activated Carbon Supported Pd Catalyst for Hydrodechlorination of 2,4-Dichlorophenol or Chloropentafluoroethane

**DOI:** 10.3390/molecules24040674

**Published:** 2019-02-14

**Authors:** Haodong Tang, Bin Xu, Meng Xiang, Xinxin Chen, Yao Wang, Zongjian Liu

**Affiliations:** Institute of Industrial Catalysis, College of Chemical Engineering, Zhejiang University of Technology, Hangzhou 300014, China; tanghd@zjut.edu.cn (H.T.); 2111601033@zjut.edu.cn (B.X.); 2111601241@zjut.edu.cn (M.X.); 2111701032@zjut.edu.cn (X.C.); 2111801033@zjut.edu.cn (Y.W.)

**Keywords:** activated carbon, hydrodechlorination, nitrogen doping, Pd

## Abstract

Nitrogen-doped activated carbon (N-AC) obtained through the thermal treatment of a mixture of HNO_3_-pretreated activated carbon (AC) and urea under N_2_ atmosphere at 600 °C was used as the carrier of Pd catalyst for both liquid-phase hydrodechlorination of 2,4-dichlorophenol (2,4-DCP) and gas-phase hydrodechlorination of chloropentafluoroethane (R-115). The effects of nitrogen doping on the dispersion and stability of Pd, atomic ratio of Pd/Pd^2+^ on the surface of the catalyzer, the catalyst’s hydrodechlorination activity, as well as the stability of N species in two different reaction systems were investigated. Our results suggest that, despite no improvement in the dispersion of Pd, nitrogen doping may significantly raise the atomic ratio of Pd/Pd^2+^ on the catalyst surface, with a value of 1.2 on Pd/AC but 2.2 on Pd/N-AC. Three types of N species, namely graphitic, pyridinic, and pyrrolic nitrogen, were observed on the surface of Pd/N-AC, and graphitic nitrogen was stable in both liquid-phase hydrodechlorination of 2,4-DCP and gas-phase hydrodechlorination of R-115, with pyridinic and pyrrolic nitrogen being unstable during gas-phase hydrodechlorination of R-115. As a result, the average size of Pd nanocrystals on Pd/N-AC was almost kept unchanged after liquid-phase hydrodechlorination of 2,4-DCP, whereas crystal growth of Pd was clearly observed on Pd/N-AC after gas-phase hydrodechlorination of R-115. The activity test revealed that Pd/N-AC exhibited a much better performance than Pd/AC in liquid-phase hydrodechlorination of 2,4-DCP, probably due to the enhanced stability of Pd exposed to the environment resulting from nitrogen doping as suggested by the higher atomic ratio of Pd/Pd^2+^ on the catalyst surface. In the gas-phase hydrodechlorination of R-115, however, a more rapid deactivation phenomenon occurred on Pd/N-AC than on Pd/AC despite a higher activity initially observed on Pd/N-AC, hinting that the stability of pyridinic and pyrrolic nitrogen plays an important role in the determination of catalytic performance of Pd/N-AC.

## 1. Introduction

Chlorinated organics are among the most significant and widespread harmful materials in the environment. They have been found in a variety of environmental situations, such as water, air, and soil. For instance, chlorophenols, a class of persistent pollutants that are toxic and resistant to environmental degradation through chemical, biological, and photolytic processes, have become an important component of industrial wastewater due to their extensive uses as raw materials or intermediates to produce pesticides, dyes, and leather [1,2,3]. Another well-known example is chlorofluorocarbons, many of which have been widely used as refrigerants, aerosol propellants, or solvents. However, they are scientifically linked to the depletion of the ozone layer and thus pose a serious threat to the global climate [4,5]. Since these chlorinated organics are very difficult to destroy by incineration, new technologies must be developed to recycle or destroy them. Catalytic hydrodechlorination has been considered as a promising treatment technique for both chlorophenols [6,7,8,9,10] and chlorofluorocarbons [11,12,13,14]. For example, liquid-phase catalytic hydrodechlorination offers a nondestructive way to dispose of chlorophenols and thus allows a recovery of the treatment product (e.g., phenol or cyclohexanone) [6,7]. In the case of treating chlorofluorocarbons, gas-phase catalytic hydrodechlorination can transform chlorofluorocarbons into hydrofluorocarbons [10,11,12], which can serve as a replacement for chlorofluorocarbons and are significantly less harmful to the environment.

Supported Pd catalysts are commonly used in the hydrodechlorination process due to their high activity and carbon materials, e.g., activated carbons (AC) are often selected as the supports because of their high specific surface area and excellent stability. During reactions, however, Pd tends to aggregate on the surface of carbon carriers and thus loses its catalytic activity, probably due to the relatively weak interaction between carbon and metal active components [14,15]. It has been reported that nitrogen doping may not only change the physical and chemical properties of carbon carriers but may also enhance the interaction between support and metal active components, and thus improve the activity and stability of the catalyst [15,16]. Indeed, many studies have shown that nitrogen doping of carbon supports can greatly improve the catalytic performance of Pd catalysts in liquid-phase catalytic hydrodechlorination [8,9,10,17,18,19]. Zhou et al. reported that nitrogen-doped graphene-supported Pd catalysts exhibited much higher catalytic activities for hydrodechlorination of 2,4-dichlorophenol (2,4-DCP) in comparison with Pd/graphene [17]. Baeza et al. found that nitrogen doping can greatly improve the performance of carbon-supported Pd catalysts in the hydrodechlorination of 4-chlorophenol to phenol [18]. In our group, we observed that nitrogen-doped mesoporous carbon prepared through a one-step method was a more effective Pd support than un-doped mesoporous carbon in the hydrodechlorination of 2,4-DCP [20]. Although much work has been done on liquid-phase catalytic hydrodechlorination, there is little work on the use of nitrogen-doped carbon supports in the gas-phase catalytic hydrodechlorination. In particular, to the best of our knowledge, a comparative study on the use of nitrogen-doped activated carbon (N-AC) in both liquid-phase catalytic hydrodechlorination and gas-phase catalytic hydrodechlorination is still missing.

**Scheme 1:**
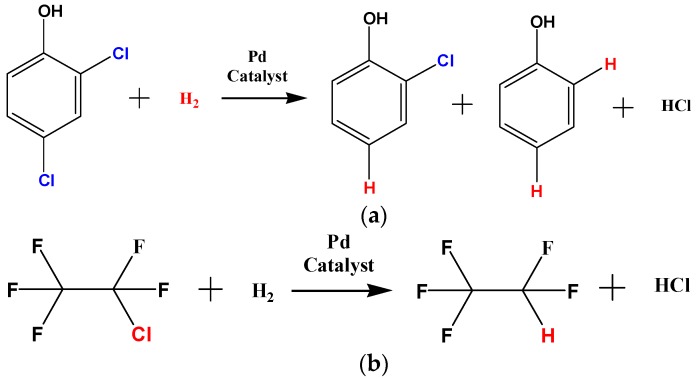
Two hydrodechlorination reactions: (**a**) hydrodechlorination of 2,4-DCP and (**b**) hydrodechlorination of R-115.

In this work, we present a comparative study on the use of N-AC supported Pd catalysts in both liquid-phase catalytic hydrodechlorination of 2,4-DCP (see Scheme 1a) and gas-phase catalytic hydrodechlorination of chloropentafluoroethane (R-115) (see Scheme 1b). The present work mainly focuses on two aspects: (i) the stability of N species in two different reaction systems and (ii) the effects of nitrogen doping on the dispersion and stability of Pd, atomic ratio of Pd/Pd^2+^ on the surface of the catalyst, and the catalyst’s hydrodechlorination activity.

## 2. Results and Discussion

### 2.1. Catalyst Characterization

**Figure 1:**
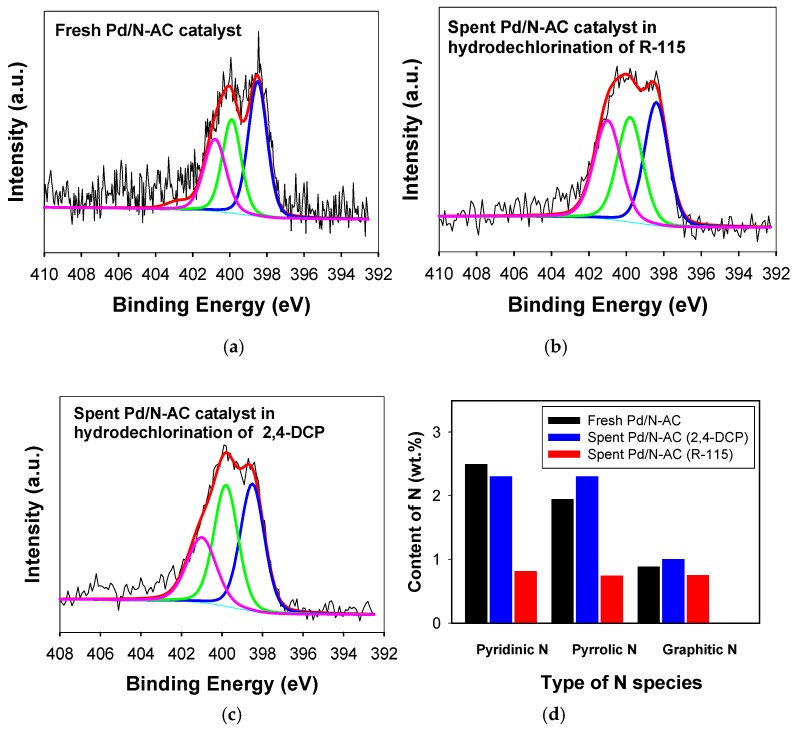
N1s XPS spectra (**a**–**c**) and the content of N of different types (**d**) of fresh Pd/N-AC catalyst and spent Pd/N-AC catalysts in hydrodechlorination of R-115 and 2,4-DCP. The raw data are shown by the black curve, and the fitting curve is indicated by the red curve. The decomposed peaks are presented by the blue (pyridinic N), green (pyrrolic N), and purple curves (graphitic N).

Nitrogen was introduced into AC through the thermal treatment of a mixture of AC and urea. In order to determine whether N species were present on the surface of catalyst, the as-prepared catalyst was characterized by X-ray photoelectron spectroscopy (XPS). The wide scan XPS spectrum of the Pd/N-AC catalyst indicates the presence of N on the surface of Pd/N-AC catalyst (see Figure S1 in the Supported Materials). Figure 1a presents the N1s XPS spectrum of Pd/N-AC catalyst. For comparison, the N1s XPS spectra of spent Pd/N-AC catalysts are also shown in Figure 1. As indicated by the decomposed peaks, three types of N were identified: pyridinic, pyrrolic, and graphitic N. Pyridinic N bonded with two C atoms as a member of a hexagon, pyrrolic N bonded with two C atoms by forming a pentagon, and graphitic-N substituted for the C atom in graphene layer by bonding to three C atoms in a sp^2^ configuration [21]. The total content of N on the surface of Pd/N-AC catalyst determined by XPS is about 5.3 wt.%, with pyridinic N being about 2.5 wt.%, pyrrolic N being about 2.0 wt.%, and graphitic N being about 0.8 wt.%, respectively (see black bars in Figure 1d). Despite the successful incorporation of nitrogen into AC, nitrogen doping seemed not to have a significant effect on the pore volume and surface area of the catalyst. The total pore volume determined by N_2_ adsorption–desorption isotherms is 0.42 m^3^/g for Pd/AC or 0.41 m^3^/g for Pd/N-AC, with the surface area obtained through Brunauer–Emmett–Teller (BET)method being about 840 m^2^/g for Pd/AC or 825 m^2^/g for Pd/N-AC.

**Figure 2:**
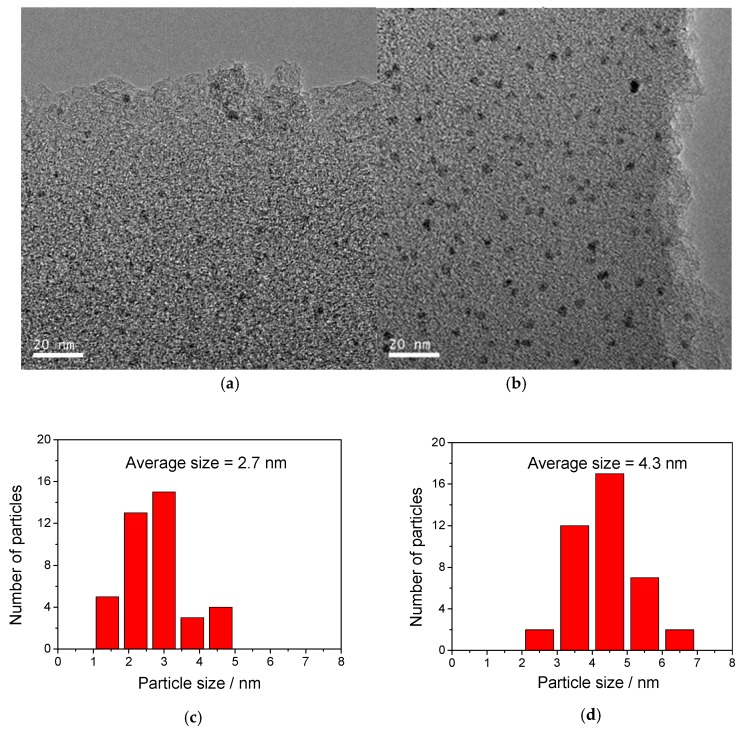
TEM images of (**a**) Pd/AC and (**b**) Pd/N-AC, and Pd size distribution of Pd/AC (**c**) and (**d**) Pd/N-AC.

**Figure 3:**
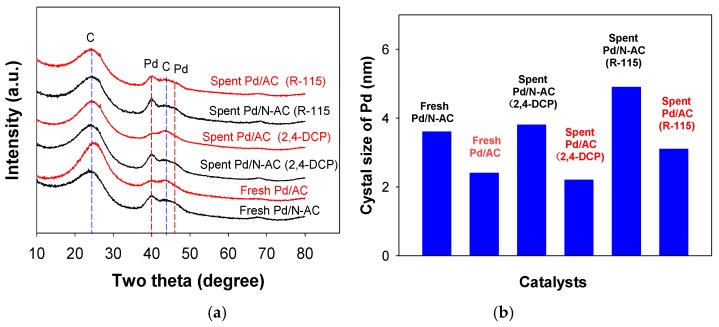
(**a**) XRD patterns of fresh and spent catalysts and (**b**) crystal size of Pd of different catalysts calculated based on the Scherrer’s equation along <111> direction.

**Figure 4:**
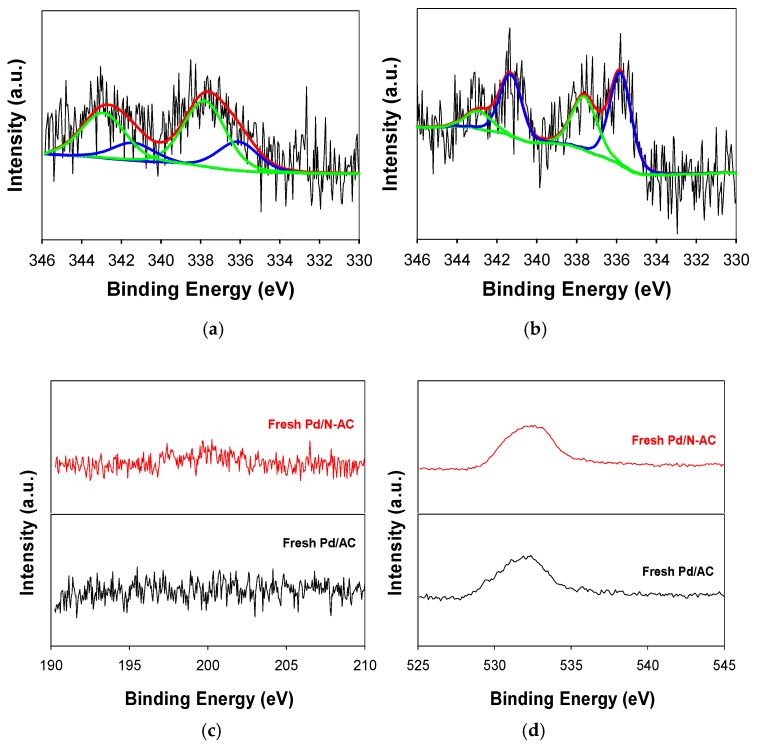
Pd 3d XPS spectra of fresh (**a**) Pd/AC and (**b**) Pd/N-AC catalysts. Cl 2p and O 1s XPS spectra of two catalysts: (**c**) Cl 2p and (**d**) O1s. The raw data are shown by the black curve, and the fitting curve is indicated by the red curve. The decomposed peaks are presented by the blue (Pd^0^) and green (Pd^2+^) curves.

The loading of Pd determined by elemental analysis is about 2.95 wt.% for Pd/AC or 2.97 wt.% for Pd/N-AC. To elucidate the effect of nitrogen-doping on the size of Pd catalyst, the microstructures of both Pd/AC and Pd/N-AC was investigated by TEM. Figure 2 presents the TEM images of the as-prepared Pd/AC and Pd/N-AC catalysts, along with the corresponding Pd size distribution of two catalysts. To our surprise, the result showed that nitrogen doping cannot improve the dispersion of Pd, with the average Pd size of Pd/AC being 2.8 nm and that of Pd/N-AC being 4.3 nm. The XRD measurement also suggested that the crystal size of Pd on Pd/AC is smaller than that on Pd/N-AC (Figure 3). It has been reported that when carbon supports are doped with nitrogen, the spin density of the supported Pd decreases remarkably with its d band center moving to a deeper energy level, thus leading to an improvement in the oxidation resistance of Pd [22]. To determine whether such an effect exists in the Pd/N-AC catalyst, Pd 3d XPS spectra of both Pd/AC and Pd/N-AC were recorded (Figure 4). An analysis of these XPS spectra indicated that the atomic ratio of Pd/Pd^2+^ on the catalyst surface is about 1.2 on Pd/AC or 2.2 on Pd/N-AC. Since Pd^2+^ can easily be reduced to Pd under H_2_ atmosphere at 200 °C, we speculated that the ionic Pd species observed should be attributed to the oxidation of Pd when the catalysts were exposed to air. This inference is supported by the Cl 2p spectra of both Pd/AC and Pd/N-AC (see Figure 4c,d), where no peak in the energy range of 190–210 eV could be observed. The O1s XPS spectra indicated that the presence of O on the surface of the catalyst (the amount of O on the surface of the fresh and spent catalysts ranges from about 4 to 8 atm.%, see Table S1 in the Supported Materials), which mainly comes from the contribution of the support (namely AC) and adsorbed O-containing materials. These observations suggest that despite no improvement in the dispersion of Pd, nitrogen doping can enhance the stability of Pd exposed to the environment and thus raise the atomic ratio of Pd/Pd^2+^ on the catalyst surface.

### 2.2. Catalytic Performance

**Figure 5:**
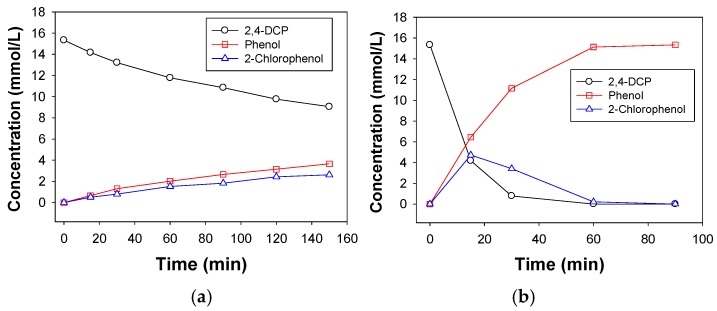
The concentrations of 2,4-DCP, 2-Chlorophenol and phenol as a function of reaction time over (**a**) Pd/AC and (**b**) Pd/N-AC.

**Figure 6:**
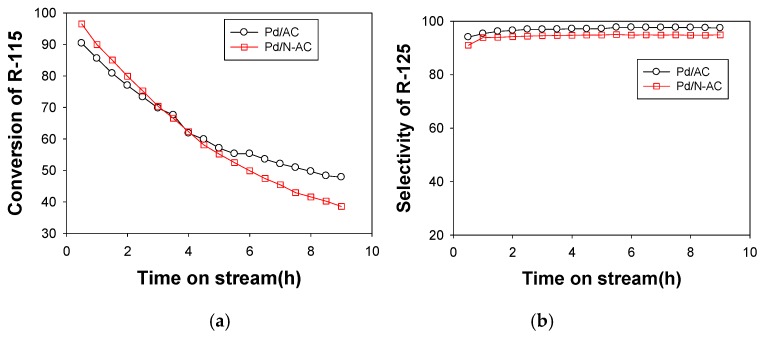
Catalytic performance of hydrodechlorination of R-115 over Pd/AC and Pd/N-AC: (**a**) R-115 conversion and (**b**) R-125 selectivity.

The catalytic performances of Pd/AC and Pd/N-AC catalysts in the hydrodechlorination of 2,4-DCP to phenol are shown in Figure 5. Under our experimental conditions, there was no deep hydrogenation product (e.g., cyclohexanone) and only 2-chlorophenol (2-CP) was identified as the dechlorinated by-product over all three catalysts. The absence of 4-chlorophenol can be ascribed to the steric hindrance, by which the para-substituted Cl was easily attacked by the active H [23]. Therefore, the possible pathway of hydrodechlorination of 2,4-DCP in our case was 2,4-DCP → 2-CP → phenol. This implies that prolonging the reaction duration favors the formation of phenol, which is consistent with the findings from Figure 4 that show that with an increasing reaction time the concentration of phenol increases monotonously. From Figure 4, we also observed that the incorporation of nitrogen into AC can greatly improve the performance of Pd catalysts. For example, the conversions of 2,4-DCP at a reaction time of 60 min over Pd/AC and Pd/N-AC catalysts are, respectively, about 29% and 99%. However, a positive doping effect cannot be observed in the gas-phase hydrodechlorination of R-115. As shown in Figure 6, while the selectivity to pentafluoroethane (R-125) over Pd/AC or Pd/N-AC catalyst has no obvious change during time on stream, deactivation can be observed on both catalysts. Despite the initial conversion of R-115 over Pd/N-AC being slightly higher than over Pd/AC, the former deactivated more rapidly than the latter. After 9 h on stream, for example, the conversion of R-115 was reduced to about 48% over Pd/N-AC and 39% over Pd/AC. These experimental results suggest that the incorporation of nitrogen into AC cannot improve the performance of Pd catalysts in the gas-phase hydrodechlorination of R-115.

**Figure 7:**
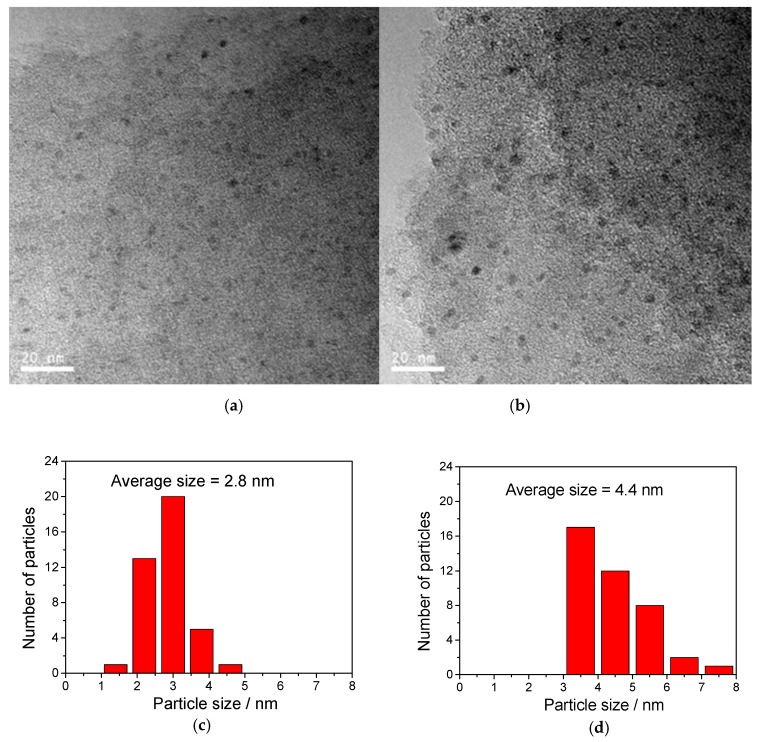
TEM images of (**a**) spent Pd/AC and (**b**) spent Pd/N-AC, and Pd size distribution of (**c**) spent Pd/AC and (**d**) spent Pd/N-AC in the hydrodechlorination of 2,4-DCP.

The above activity test indicates that the effect of nitrogen doping on the catalytic performance seems to be different in two hydrodechlorination reaction systems. In our previous study on hydrodechlorination of 2,4-DCP over Pt/MC catalyst [20], we found that during reaction the crystal growth of Pd occurred and nitrogen doping could enhance the resistance to the growth of nanosized Pd. To determine whether a similar phenomenon could be observed on Pt/AC catalysts, we examined the spent catalysts by TEM (Figure 7). In comparison with the results shown in Figure 2, it is clear that the size of Pd particles in both Pt/AC and Pt/N-AC catalysts has no obvious change after reaction, which is consistent with the measurement by XRD (see Figure 3). Thus, the better catalytic performance observed on Pd/N-AC than on Pd/AC in the hydrodechlorination of 2,4-DCP cannot be attributed to the better Pd dispersion and higher resistance to the growth of nanosized Pd under reaction conditions. Additionally, despite the fact that after reaction, the Pd loading reduced to 2.74 wt.% on Pd/AC and 2.85 wt.% on Pd/N-AC, the loss of Pd during reaction was not responsible for the observed difference in activity between the two catalysts because the lost Pd was still in the reactor and thus participated in the reaction. Since the hydrodechlorination of 2,4-DCP was carried out in water, it was expected that the following two aspects might be responsible for the positive effect on the performance of Pd catalysts via nitrogen doping: (i) Nitrogen doping can improve resistance to oxidation of Pd in water during liquid-phase hydrodechlorination of 2,4-DCP as suggested by the result that nitrogen doping can significantly raise the atomic ratio of Pd/Pd^2+^ on the catalyst surface and (ii) The presence of pyridinic and pyrrolic nitrogen species on the support surface may improve the hydrophilicity of Pd/N-AC, which favors the reactant molecules in the water accessing the active sites in the pores via capillary action and thus raises the catalytic performance of the catalysts.

**Figure 8:**
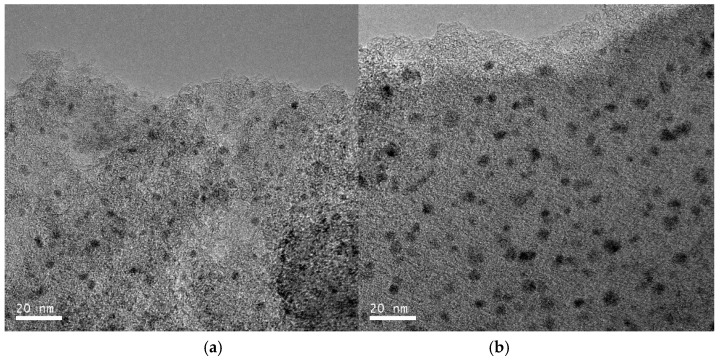

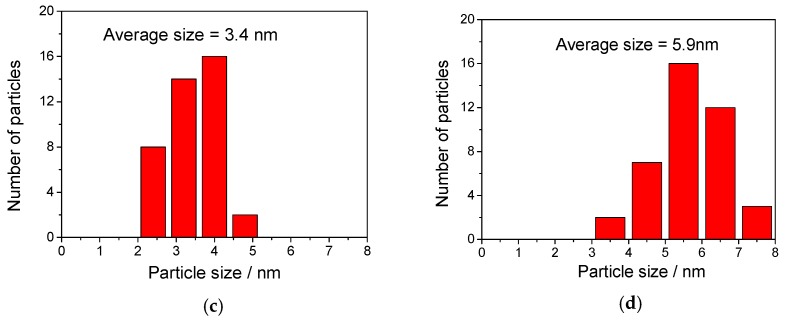
TEM images of (**a**) spent Pd/AC, and (**b**) spent Pd/N-AC, and Pd size distribution of (**c**) spent Pd/AC and (**d**) spent Pd/N-AC in the hydrodechlorination of R-115.

In the gas-phase hydrodechlorination of R-115, however, the TEM investigation suggests that the crystal growth of Pd during reaction occurs on both Pt/AC and Pt/N-AC catalysts due to a high reaction temperature (Figure 8). After 9 h time on stream the size of Pd particles was raised from 2.7 to 3.4 nm (about a 25.9% increase) over Pd/AC and from 4.3 to 5.9 nm (about a 37.2% increase) over Pd/N-AC. Thermodynamically, the driving force for crystal growth is the minimization of free energy in the system, and a high reaction temperature may promote the diffusion of Pd atoms or clusters and thus favors the growth of nanosized Pd particles. XRD measurement also confirmed the crystal growth of Pd during the reaction (Figure 3). Additionally, since there was only a slight change in the Pd loading of two catalysts after the reactions were observed (2.85 wt.% on Pd/AC and 2.82 wt.% on Pd/N-AC), the deactivation phenomenon cannot be attributed to the loss of Pd during reaction. Therefore, the sintering of Pd particles should be the main reason for the deactivation of two catalysts during time on stream, and the result that Pd/N-AC deactivates more rapidly than Pd/AC can be attributed to a higher growth rate of Pd on Pd/N-AC. To understand the reason for the higher growth rate of Pd observed on Pd/N-AC catalyst, the stability of N species during reaction was investigated by XPS (Figure 1b,c). When compared with the fresh Pd/N-AC catalyst with about 2.5 wt.% pyridinic N, 2.0 wt.% pyrrolic N, and 0.8 wt.% graphitic N (Figure 1b), the spent Pd/N-AC catalyst for hydrodechlorination of R-115 possesses almost the same amount of graphitic nitrogen (about 0.8 wt.%) but much less pyridinic (about 0.8 wt.%) and pyrrolic nitrogen (about 0.7 wt.%). In the hydrodechlorination of 2,4-DCP, however, the amounts of three types of N species on the spent Pd/N-AC catalyst are very close to those on the fresh catalyst. These observations hint that graphitic nitrogen is stable in both hydrodechlorination of 2,4-DCP and R-115, with pyridinic and pyrrolic nitrogen being unstable during hydrodechlorination of R-115. Thus, we speculate that a higher growth rate of Pd observed on Pd/N-AC catalyst should be related to the loss of surface nitrogen (namely pyridinic and pyrrolic nitrogen), which may lead to the instability of Pd particles. As a result, enhancing the stability of both pyridinic and pyrrolic nitrogen is crucial for the nitrogen-doped carbon materials used in the gas-phase catalytic hydrodechlorination.

## 3. Materials and Methods

### 3.1. Materials and Chemicals

All chemicals were of analytical grade and used as received. Except for PdCl_2_ (purchased from Sino-Platinum Metals Co. Ltd., Kunming, China) and nitric acid (purchased from Shanghai Lingfeng Chemical Reagent Co., Ltd., Shanghai, China), all other reagents were obtained from Aladdin Chemical Reagent Co., Ltd (Shanghai, China). Coconut shell AC was purchased from Hainan Coconut Shell Active Carbon Plant (Hainan, China).

### 3.2. Preparation of N-AC 

Coconut shell AC was crushed and sieved to granules in the range of 40–60 mesh. These AC granules were then washed with deionized water and dried at 110 °C for about 24 h. Thirty grams of AC (40–60 mesh) was put into a flask containing 100 mL of 20% dilute nitric acid. The flask was then put in a water bath (90 °C), and the AC-nitric acid mixture was refluxed for 5 h before cooling to room temperature and filtering. Finally, the residue (namely AC) was washed with deionized water until neutral pH eluate was obtained and dried at 110 °C for about 8 h.

The HNO_3_-pretreated AC (2 g) was mixed with 2.5 g of urea and then put into a tubular furnace. Before heating, N_2_ atmosphere (30 mL/min) was passed through the tubular furnace for 30 min to remove the air. After turning off the N_2_ flow, the HNO_3_-pretreated AC was heated at 10 °C/min to 600 °C and then kept at this temperature for 3 h. Finally, N-AC was obtained after the tubular furnace was cooled to room temperature in a N_2_ atmosphere (30 mL/min). 

### 3.3. Preparation and Reduction of Supported Pd Catalysts 

Supported Pd catalysts were prepared through a wet impregnation method. Briefly, 1.00 g support was placed into a container, and then 2.50 mL of PdCl_2_ solution (20.31 g/L) was added dropwise to the support, followed by impregnation for 12 h. Supported Pd catalysts were reduced by H_2_ at 200 °C for 2 h before the hydrodechlorination activity test and characterization.

### 3.4. Characterization

Elemental analysis was conducted on an elemental analyzer (Vario Macro cube, Germany). Surface areas of the samples were measured on a Micrometrics ASAP 2020 (Micrometrics Instrument Co., Norcross, GA) instrument. The samples were degassed under N_2_ flow at 200 °C for 6 h prior to analysis at –196 °C (77 K). X-ray diffraction (XRD) patterns of the samples were obtained using a Rigaku D/max-RA powder diffraction-meter (Rigaku, Tokyo, Japan) equipped with Cu Ka radiation. The Pd contents in the catalysts were determined on a 7600 ultraviolet spectrophotometer. Transmission electron microscopy (TEM) investigation of the samples was conducted on a JEOL JEM-1200EX electron microscope (JEOL Co., Tokyo, Japan). The average particle size of Pd particles was calculated by Image J software. X-ray photoelectron spectroscopy (XPS) was performed on aKratos AXIS Ultra DLD using a monochromatic Al Ka excitation source (1486.6 eV). The C 1s peak (284.6 eV) was used as the internal standard.

### 3.5. Catalyst Activity Test

The hydrodechlorination of R-115 was carried out at 450 °C in a fixed-bed reactor with an inner diameter of 15 mm. The loading of catalyst was 2 mL, the flow rates of R-115 and H_2_ were, respectively, 5 and 15 mL/min, and the space velocity was 600 h^−1^. Before conducting online analysis by gas chromatograph (Jiedo GC-1690), the hydrodechlorination product was washed by sodium hydroxide solution and dried by anhydrous calcium chloride.

The hydrodechlorination of 2,4-DCP was conducted in a three-necked flask (250 mL) under atmospheric pressure. The flask, which contains 0.10 g of catalyst suspended in 200 mL of 2,4-DCP solution (containing 0.5 g 2,4-DCP) with a pH of 12 adjusted by 1.0 M NaOH, was placed in a water-bath (SDC-6, Scientz Co, China) with a temperature of 25 ± 0.5 °C. The suspension was purged with a N_2_ flow (50 mL min1) for 30 min, and then the N_2_ flow was switched to a H_2_ flow (180 mL/min) under continuous vigorous stirring (850 rpm). Samples were taken at 30 min intervals and the catalyst particles were removed by fast filtration. The concentrations of the reactant, intermediate, and product in the filtrate were determined by a gas-phase chromatography with a flame ionization detector. Prior to gas-phase chromatography analysis, the filtrate was neutralized using 0.1 M HCl solution. 

## 4. Conclusions

In summary, we have presented a comparative study on the use of N-AC supported Pd catalysts in both liquid-phase catalytic hydrodechlorination of 2,4-DCP and gas-phase catalytic hydrodechlorination of R-115. XPS investigation suggests that graphitic, pyridinic, and pyrrolic nitrogen were observed on the surface of Pd/N-AC, and while the former is stable in both liquid-phase hydrodechlorination of 2,4-DCP and gas-phase hydrodechlorination of R-115, the latter two are unstable during gas-phase hydrodechlorination of R-115. As a result, the average size of Pd nanocrystals on Pd/N-AC was almost unchanged after liquid-phase hydrodechlorination of 2,4-DCP, whereas crystal growth of Pd was clearly observed on Pd/N-AC after gas-phase hydrodechlorination of R-115. The activity test revealed that Pd/N-AC exhibits a better performance than Pd/AC in liquid-phase hydrodechlorination of 2,4-DCP probably due to the enhanced stability of Pd exposed to the environment resulting from nitrogen doping as suggested by the higher atomic ratio of Pd/Pd^2+^ observed on the catalyst surface of Pd/N-AC. In the gas-phase hydrodechlorination of R-115, however, a more rapid deactivation occurs on Pd/N-AC than on Pd/AC, hinting that the stability of pyridinic and pyrrolic nitrogen plays an important role in the determination of catalytic performance of Pd/N-AC.

## Supplementary Materials

The Supplementary Materials are available online.
