# Supplementary material for: Catalytic Performance of Nitrogen-Doped Activated Carbon Supported Pd Catalyst for Hydrodechlorination of 2,4-Dichlorophenol or Chloropentafluoroethane
Source: Molecules. 2019 Feb 14;24(4):674. doi: 10.3390/molecules24040674 (PMC6413172; doi:10.3390/molecules24040674)
Supplement: Supplementary file 1 [file molecules-24-00674-s001.pdf]

Catalytic Performance of Nitrogen-Doped Activated Carbon Supported Pd Catalyst for Hydrodechlorination of 2,4-Dichlorophenol or Chloropentafluoroethane

Haodong Tang [1], Bin Xu[1], Meng Xiang[1], Xinxin Chen[1], Yao Wang[1] and Zongjian Liu[1,*]

[1] Institute of Industrial Catalysis, College of Chemical Engineering, Zhejiang University of Technology, Hangzhou, 300014, P.R. China

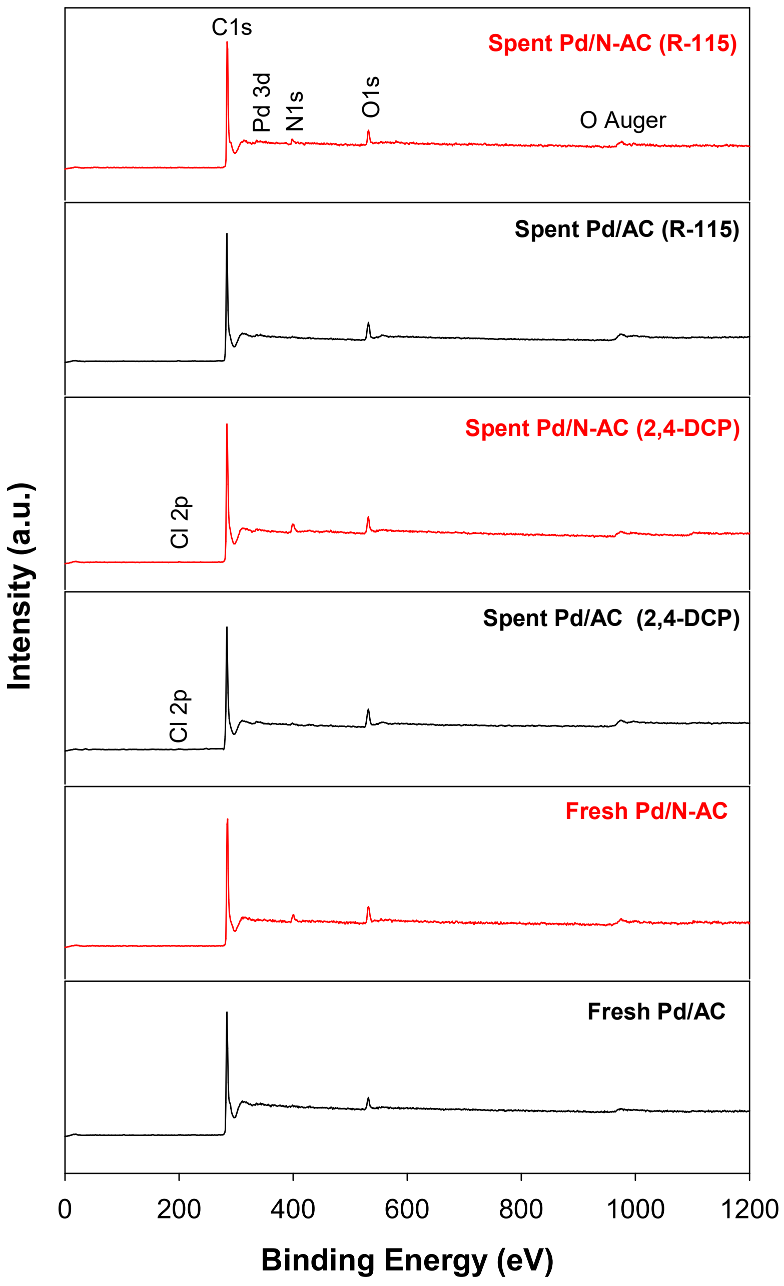

Fig. 1S Wide-scan XPS spectra of fresh and spent catalysts.

Table 1S The concentration of O and Cl on the surface of catalysts before and after reaction determined by the wide-scan XPS spectra *

Sample	Content of O/%	Content of Cl/%
fresh Pd/AC-	4.3	-
fresh Pd/N-AC	5.9	-
spent Pd/AC (2,4-DCP)	7.3	0.4
spent Pd/N-AC (2,4-DCP)	6.9	0.5
spent Pd/AC (R-115)	6.8	-
spent Pd/N-AC (R-115)	5.0	-

* The observed O mainly comes from the support (namely AC) and adsorbed O-containing materials. The Cl observed on the surface of the catalysts after hydrodechlorination of 2,4-DCP can be attributed to the adsorption of the product HCl by the catalysts.
